# Investigating the Molecular Mechanisms of the Anticancer Effects of Eugenol and Cinnamaldehyde Against Colorectal Cancer (CRC) Cells In Vitro

**DOI:** 10.3390/ijms27020649

**Published:** 2026-01-08

**Authors:** Alberto Bernacchi, Maria Chiara Valerii, Renato Spigarelli, Nikolas Kostantine Dussias, Fernando Rizzello, Enzo Spisni

**Affiliations:** 1 Department of Biological, Geological, and Environmental Sciences (BiGeA), University of Bologna, Via Selmi 3, 40126 Bologna, Italy; alberto.bernacchi2@unibo.it (A.B.); mariachiara.valerii2@unibo.it (M.C.V.); enzo.spisni@unibo.it (E.S.); 2 Inflammatory Bowel Disease (IBD) Unit, Istituti di Ricovero e Cura a Carattere Scientifico (IRCCS), Azienda Ospedaliero-Universitaria di Bologna, University of Bologna, Via Massarenti 9, 40138 Bologna, Italy; nikolas.dussias@studio.unibo.it (N.K.D.); fernando.rizzello@unibo.it (F.R.); 3 Department of Medical and Surgical and Sciences, University of Bologna, Via Massarenti 9, 40138 Bologna, Italy

**Keywords:** colorectal cancer, eugenol, cinnamaldehyde, essential oils, antitumor activity, transcriptomics

## Abstract

Colorectal cancer is one of the leading causes of cancer-associated mortality, and multifactorial resistance remains one of the main challenges in its treatment. Essential oils and their main compounds show interesting anticancer properties, but their mechanism of action is yet to be defined. This study aims to assess the cytotoxic effects of eugenol (EU) and cinnamaldehyde (CN) on colorectal cancer (CRC) cells, highlighting possible mechanisms of action. These compounds were tested on normal immortalized colonocytes (NCM-460) and two CRC cell lines: Caco-2, a human colon epithelial adenocarcinoma cell line, and SW-620, colon cancer cells derived from a lymph node metastatic site. The efficacy of EU and CN was evaluated through CellTiter-Glo^®^ and clonogenic assays and by determining proinflammatory cytokine secretion. Transcriptome analysis was used to identify possible pathways affected by EU and CN treatments. The results confirmed that EU and CN were selectively cytotoxic and pro-apoptotic against CRC cells, with different putative mechanisms. While EU drove cytotoxicity through robust transcriptional remodeling, CN yielded a stronger anti-inflammatory action. We confirmed that EU and CN are promising natural candidates in CRC prevention and treatment, even in association with chemotherapeutic drugs.

## 1. Introduction

Colorectal cancer (CRC) is the third-most diagnosed cancer and represents the second leading cause of cancer-associated mortality. Each year, it accounts for 1.8 million new cases and nearly 900,000 deaths worldwide [1]. CRC accounts for approximately 10% of all annually diagnosed cancer cases, exhibiting a high prevalence in industrialized countries. A rising incidence of CRC is also observed in developing countries, primarily attributed to evolving lifestyle patterns and the adoption of a Western-style diet [2]. CRC exhibits significant genetic and molecular heterogeneity, driven by mutations in critical genes (*APC*, *KRAS*, *TP53*) and by dysregulation of different key pathways (Wnt, PI3K/AKT) [2,3]. These alterations lead to increased proliferation and decreased apoptosis and cell differentiation, thus contributing to tumor growth. CRC pathogenesis also involves alteration in pathways related to DNA repair and epigenetic modifications, such as histone chemical alterations, which further promote malignancy and chemoresistance [4,5,6,7]. Moreover, there is growing evidence that the tumor microenvironment, including gut microbiota and chronic low-grade gut inflammation, may enhance the oncogenic processes and tumor aggressiveness [4,6]. CRC typically arises from adenomatous polyps through a slow malignant transformation process requiring years. Polyp size typically correlates with malignant outcome, with lesions exceeding 1–2 cm associated with a significantly higher risk [2,8]. Despite advancements in diagnostic techniques and therapeutic approaches, CRC continues to exhibit high mortality rates, particularly in advanced stages, with a recurrence rate of 20–35% after treatment. However, screening programs, such as colonoscopy or fecal blood tests, have proved to be effective in reducing mortality by allowing early treatment. Prognosis in CRC is closely tied to the stage at diagnosis, with a five-year survival rate exceeding 90% for localized cases but dropping below 15% for metastatic disease [3,8,9,10].

CRC therapies involve surgery, chemotherapy, and emerging targeted biological therapies against specific molecular targets, such as anti-epidermal growth factor receptor (EGFR) and anti-vascular endothelial growth factor (VEGF). Despite these advancements, therapy resistance remains one of the major challenges in managing the disease. Drug resistance can be induced by the tumor microenvironment and by mutations within critical signaling cascades, such as the PI3K/AKT and Wnt pathways [11,12]. In this context, prevention remains a key strategy, particularly in non-sporadic forms, to avoid the progression of precancerous lesions. Given the long latency period between benign dysplasia and malignant adenocarcinoma, there is significant interest in strategies capable of decelerating this oncogenic transformation [13]. Essential oils (EOs) have garnered increasing interest in CRC therapy due to their ability to decrease colorectal cancer cell proliferation and to overcome drug resistance, thus enhancing the efficacy of traditional chemotherapies [13,14,15]. Our previous studies have demonstrated that eugenol (EU) and cinnamaldehyde (CN), single compounds of EO, exert significant anticancer effects in CRC cell lines, specifically Caco-2 and SW-620 [16]. We found that EU primarily induces late apoptosis and necrosis, while CN triggers cell cycle arrest and also enhances necrosis. Notably, both compounds demonstrated minimal effects on a normal colon mucosa cell line (NCM-460), suggesting a selective activity against CRC cells [16]. The present study aimed to further dissect the anti-tumor activity of these compounds, focusing on the molecular mechanism involved. In particular, we better characterized their efficacy by using tumor 3D spheroid models, as well as by analyzing the secretion of key cytokines involved in CRC progression, while the molecular mechanisms underlying their antitumor activity were investigated through exploratory transcriptomic and proteomic analyses.

## 2. Results

### 2.1. Effects of EU and CN on Spheroid Growth

**Figure 1:**
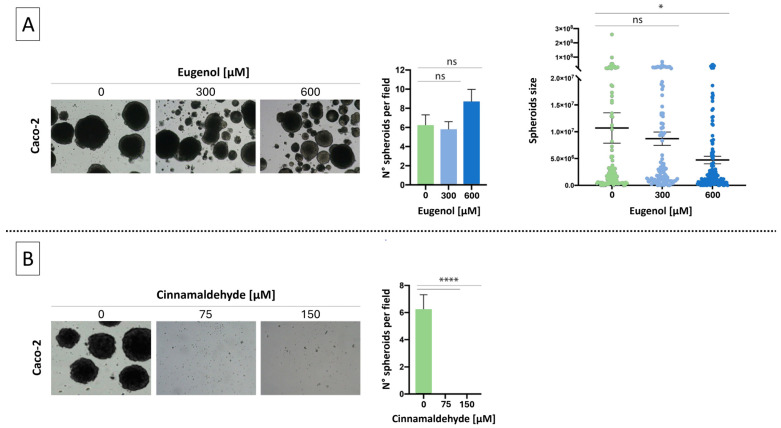
Evaluation of colony formation after treatment with EU (300 µM and 600 µM, panel (**A**)) and CN (75 µM and 150 µM, panel (**B**)). After a 14-day incubation, colony formation by Caco-2 cells were evaluated by microscopy and the number and size of colonies was determined using ImageJ software (version 1.54). Data represent the mean ± SEM of three independent experiments (n = 3). * = *p* < 0.05 and **** *p* < 0.0001 compared to untreated cells (0); ns = not statistically significant.

To evaluate the antitumor potential of EU and CN in a 3D model, we tested their effects on Caco-2 spheroid growth. Based on their non-toxic dosage previously defined in healthy NCM-460 cells [16], we selected EU 300 µM and 600 µM and CN 75 µM and 150 µM for this assay. Incubation of Caco-2 spheroids with EU (300 or 600 µM) did not result in a significant reduction in their number, although a decrease in spheroid size was clearly observed (Figure 1A). In contrast, Caco-2 cells exhibited complete inhibition of spheroid growth following CN treatment at both concentrations tested (75 and 150 µM) (Figure 1B).

### 2.2. Effect of EU and CN Co-Administration on the Metabolic Activity of CRC Cells

**Figure 2:**
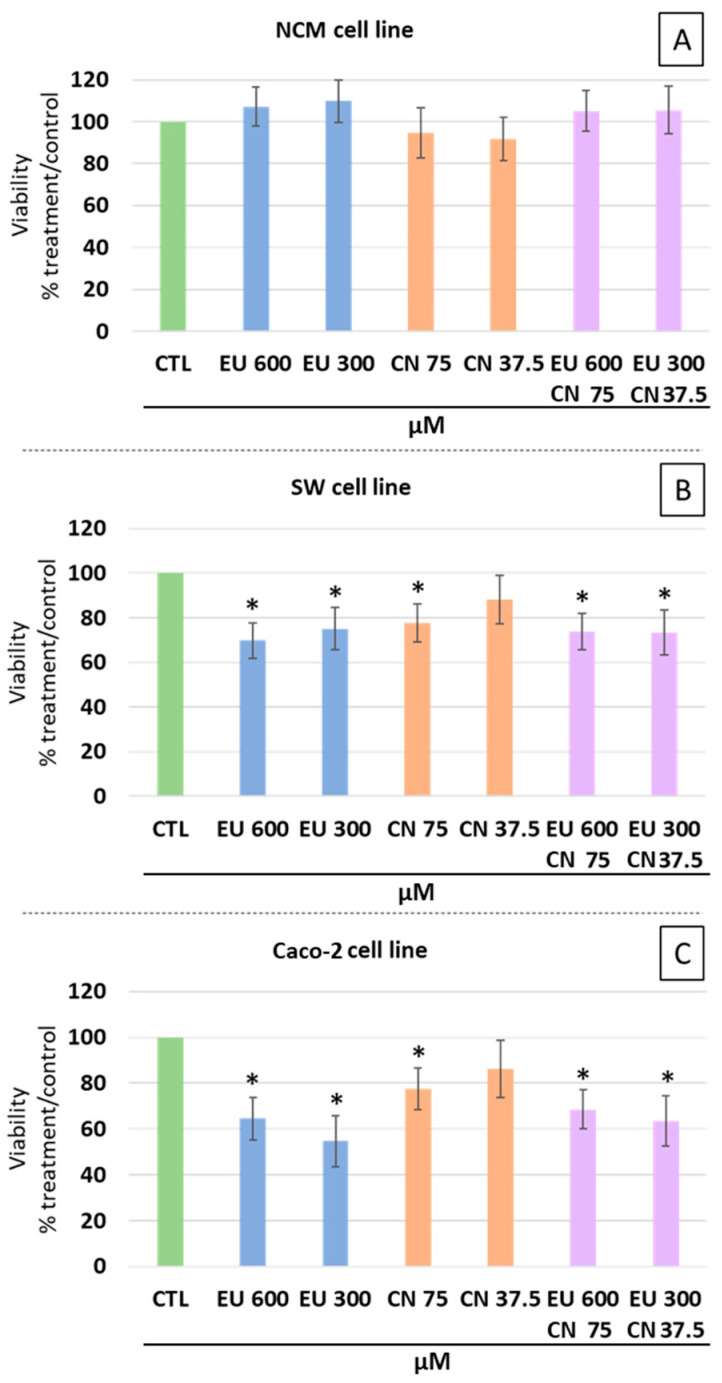
Antiproliferative and cytotoxic effects of EU and CN, alone or in combination, in NCM-460 (**A**), SW-620 (**B**), and Caco-2 (**C**) cell lines. Cells were treated with EU (600 and 300 µM) and CN (75 and 37.5 µM), alone or in combination, for 24 h and subsequently subjected to CellTiter Glo^®^ 2.0 assay. Cell viability is expressed as the percentage of untreated control cells (CTL). Data represent the mean ± SEM of three independent experiments (n = 3), each analyzed in technical triplicate. Statistical significance: * = *p* < 0.05 compared to CTL values.

Based on the growth inhibition observed in the 3D model, we moved to a 2D monolayer system to quantify the cytotoxic impact and explore the potential combined effects of the two compounds. The cytotoxic/antiproliferative potential of EU and CN, alone and in combination, was evaluated in SW-620 and Caco-2 cells, as well as in non-tumoral NCM-460 cells. In SW-620 cells, EU significantly reduced viability by 30.2% at 600 µM and 24.9% at 300 µM. Similarly, CN at 75 µM significantly affected SW-620 cell viability (−22.3%), while CN at 37.5 µM had no effect. A statistically significant reduction in cell proliferation was also observed for the combined treatments with respect to control cells (Figure 2B): EU 600 µM + CN 75 µM (−26.2%); EU 300 µM + CN 37.5 µM (−26.7%). These effects were similar to those obtained with single treatments. In Caco-2 cells, EU at 600 µM and 300 µM resulted in significantly reduced viabilities, with decreases of 35.4% and 45.3%, respectively, comparable to reductions underlying the absence of a dose-response effect. In the same cells, CN at 75 µM induced a significant reduction in proliferation (−22.5%), whereas at the lower concentration (37.5 µM) no anti-proliferative activity was evident. Combinations of EU and CN significantly affected Caco-2 viability (Figure 2C), with reductions of 31.4% for EU 600 µM + CN 75 µM and of 36.5% for EU 300 µM + CN 37.5 µM, values not statistically different from those obtained with the single compounds. Interestingly, none of the treatments affected the metabolic activity of NCM-460 cells (Figure 2A).

### 2.3. Cytokines Analysis

**Figure 3:**
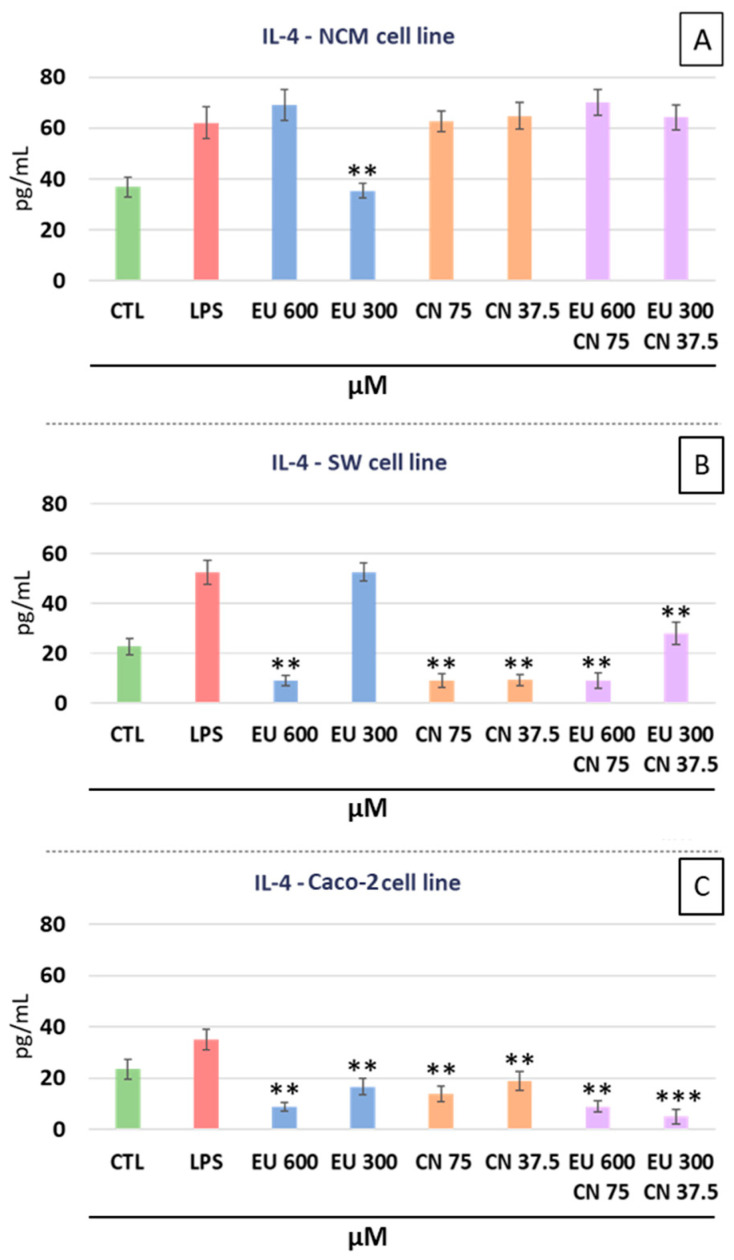
Effects of EU and CN, alone or in combination, on the secretion of the inflammatory cytokine IL-4 into the culture media. NCM-460 cells (**A**), SW-620 (**B**), and Caco-2 (**C**). ** *p* < 0.01 and *** *p* < 0.001 compared to the LPS-stimulated positive control. Data represent the mean ± SEM of three independent experiments (n = 3), with each condition analyzed in technical quadruplicate.

Since chronic inflammation is a key driver of colorectal cancer progression, we investigated whether the observed antiproliferative effects were associated with a modulation of pro-inflammatory cytokine secretion. In SW-620 cells, only EU at the higher concentration (600 µM) significantly reduced IL-4 levels (−83.17% compared to LPS positive control, *p* < 0.01). Also, CN at both the concentrations tested, reduced IL-4 levels (−83.1% for CN 75 µM, *p* < 0.01 and −82.5% for CN 37.5 µM, *p* < 0.01). The combination of EU + CN (Figure 3B) resulted in similar IL-4 reduction with respect to the LPS positive control (−83.7% for EU 600 µM + CN 75 µM, *p* < 0.01 and −46.8% for EU 300 µM + CN 37.5 µM, *p* < 0.01), showing no statistically significant difference compared with the single treatments. In Caco-2 cells (Figure 3C), EU significantly reduced IL-4 levels with respect to the LPS positive control (−74.6% for EU 600 µM, *p* < 0.01 and −52.5% for EU 300 µM, *p* < 0.01). Also, CN significantly decreased IL-4 levels in Caco-2 cells with respect to the LPS positive control (−60.4% for CN 75 µM, *p* < 0.01 and −46.2% for CN 37.5 µM, *p* < 0.01). The combination EU + CN at the higher doses (EU 600 µM + CN 75 µM) showed a reduction in IL-4 (−74.6% with respect to LPS positive control, *p* < 0.01) not statistically different from the effects observed with the single doses, while at lower doses (EU 300 µM + CN 37.5 µM) the combination EU + CN showed a more powerful effect than single doses (−85.4% with respect to LPS positive control, *p* < 0.001;). In NCM-460 cells, only EU at the lower dose (300 µM) significantly reduced IL-4 secretion with respect to LPS treatment (−43.2%, *p* < 0.01). No significant reductions were observed for CN and for the combined treatments (Figure 3A).

**Figure 4:**
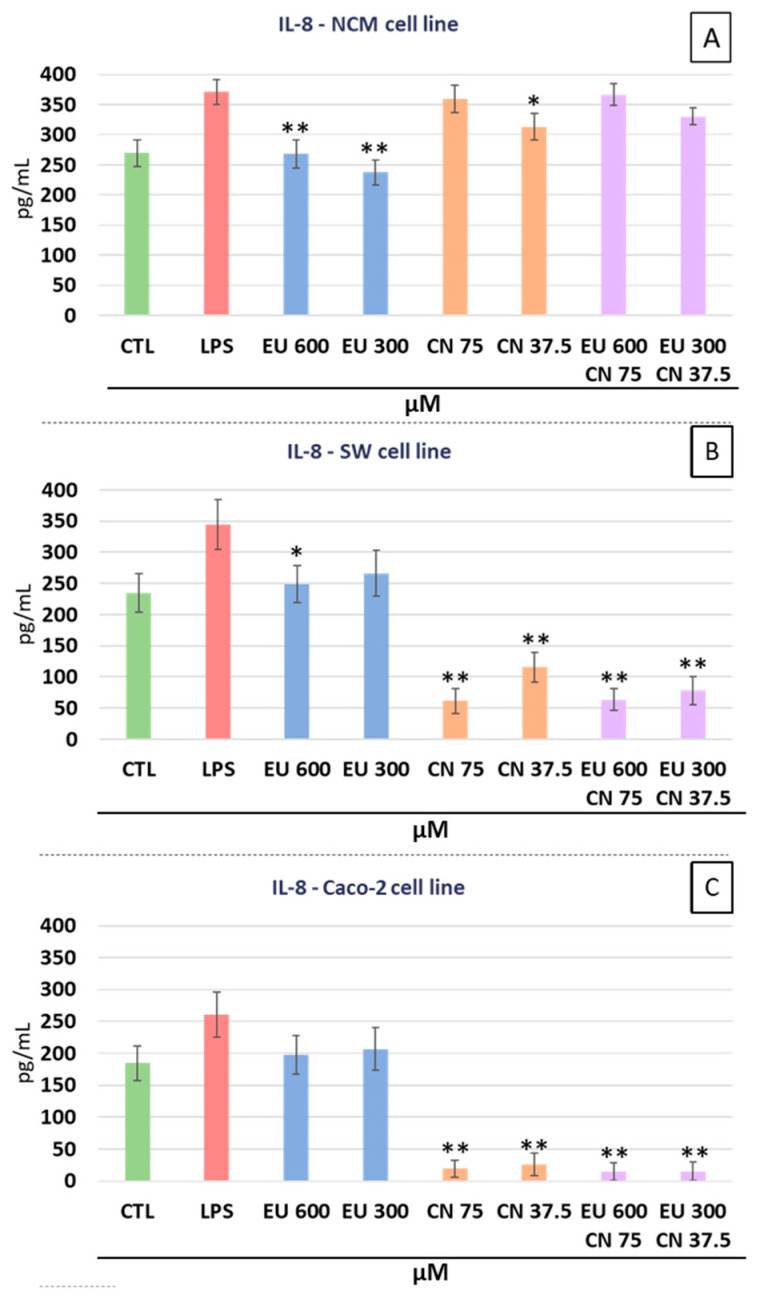
Effects of EU and CN, alone or in combination, on the secretion of the inflammatory cytokine IL-8 into the culture media. NCM-460 (**A**), SW-620 (**B**), and Caco-2 (**C**) cells. * *p* < 0.05 and ** *p* < 0.01 compared to the LPS stimulated positive control. Data represent the mean ± SEM of three independent experiments (n = 3), with each condition analyzed in technical quadruplicate.

For IL-8 secretion, in SW-620 cells (Figure 4B) a significant reduction compared to LPS positive control was observed for EU 600 µM (−27.9%, *p* < 0.05) but not for EU 300 µM. Instead, CN at both concentrations significantly and markedly reduced IL-8 levels (−82.2% for CN 75 µM, *p* < 0.01 and −66.5% for CN 37.5 µM, *p* < 0.01). In SW-620, the combined EU + CN treatment resulted in IL-8 reductions with respect to LPS treatment like those observed for CN alone (−81.6% for EU 600 µM + CN 75 µM, *p* < 0.01 and −77.3% for EU 300 µM + CN 37.5 µM, *p* < 0.01). In Caco-2 cells (Figure 4C), IL-8 secretion was not affected by EU but was significantly decreased by CN treatment with respect to LPS positive control (−92.5% for CN 75 µM, *p* < 0.01 and −90% for CN 37.5 µM, *p* < 0.01). The combination EU + CN led to IL-8 reduction with respect to LPS positive control, comparable to that obtained by CN treatment (−94.3% for EU 600 µM + CN 75 µM, *p* < 0.01 and −94.2% for EU 300 µM + CN 37.5 µM, *p* < 0.01). In NCM-460 cells (Figure 4A), both EU 600 µM and EU 300 µM significantly but moderately reduced IL-8 levels with respect to LPS positive control (−27.7% for EU 600 µM, *p* < 0.01 and −36.4% for EU300 µM, *p* < 0.01). Also, CN, but only at 37.5 µM, significantly reduced IL-8 secretion with respect to LPS treatment (−15.5% for 37.5 µM, *p* < 0.05), although the reduction was moderate. No significant effects on IL-8 were observed in NCM-460 with EU + CN combined treatment.

### 2.4. Transcriptome Analysis

**Figure 5:**
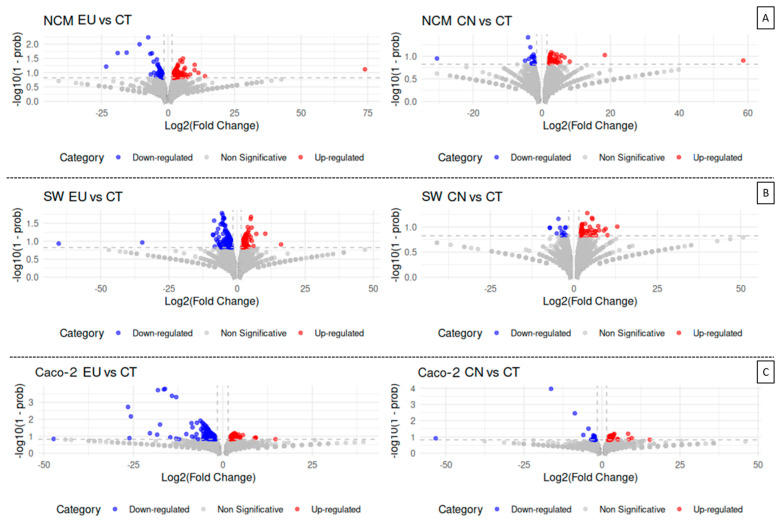
Volcano plots of differentially expressed genes after EU (600 µM) and CN (75 µM) treatment in NCM-460 (**A**), SW-620 (**B**), and Caco-2 (**C**). Each plot represents the results of the comparison between treated and untreated cells, with the *X*-axis depicting the Log2 (fold change) and the *Y*-axis showing statistical significance as −log10 (1-prob). Blue and red dots represent downregulated and overexpressed genes, respectively, with an absolute fold change > 1.5 and a posterior probability (P) > 0.85 (as determined by the NOISeq algorithm). These values were selected as thresholds, represented by the dashed gray lines; gray dots represent genes that did not exceed these significance criteria.

To gain a better understanding of the molecular mechanisms and gene networks affected by EU and CN treatments, we performed a global transcriptomic analysis using RNA sequencing on both tumoral and non-tumoral cells. For the transcriptional responses of the three cell lines (SW-620, Caco-2, and NCM-460) treated with CN (75 µM) and EU (600 µM), volcano plots (Figure 5) were generated to highlight differentially expressed genes with an absolute fold change > 1.5 and a posterior probability > 0.85 (red dots represent overexpressed genes and blue dots downregulated ones).

For SW-620 cells treated with EU, the volcano plot showed a robust transcriptional response, balanced between up- and downregulated genes. EU displays stronger transcriptional changes with respect to CN, especially for the downregulated gene cluster (Figure 5B).

For Caco-2 cells treated with EU, the volcano plot showed a clear prevalence of downregulated genes with very high absolute fold change values. The population of upregulated genes was smaller and less pronounced in terms of magnitude of fold change. Conversely, in this cell line, CN treatment showed an increased presence of the upregulated genes; however, in this case most of the differentially expressed genes exhibited fold change values close to the 1.5 threshold, suggesting a relatively milder transcriptional response for CN treatment (Figure 5C).

For NCM-460 cells treated with EU and CN, the volcano plots showed a less pronounced transcriptional response compared to the SW-620 and Caco-2 cell lines. Even in these non-transformed cells, there are few genes severely up- or downregulated by the treatments (Figure 5A).

### 2.5. NCM-460—Overrepresentation Analysis

**Figure 6:**
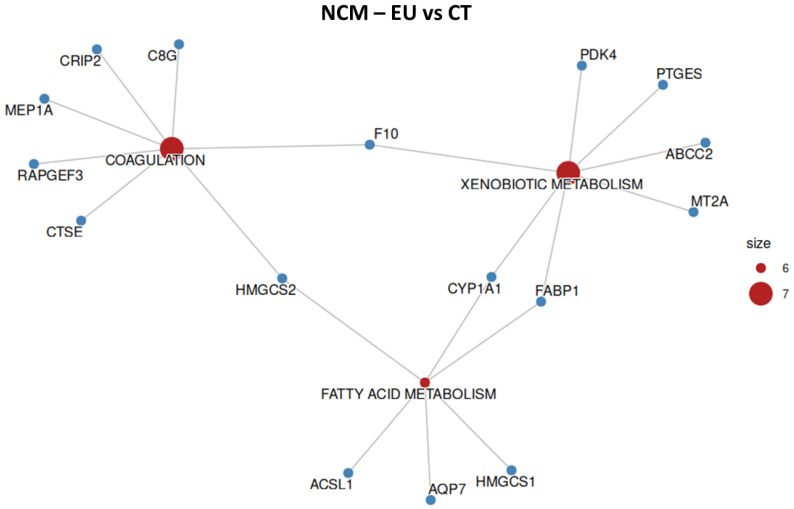
Overrepresented hallmark gene sets and respective altered genes in NCM-460 cell line when treated with EU. Size represents the number of altered genes within the groups, indicated in red.

**Figure 7:**
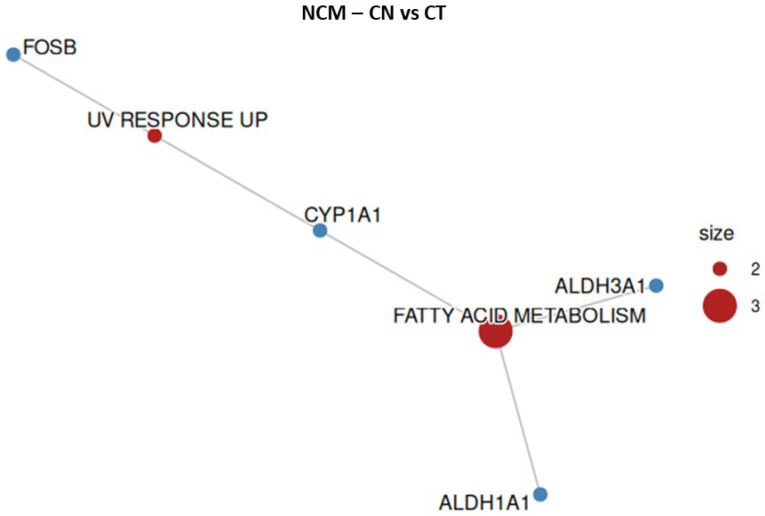
Overrepresented hallmark gene sets and respective altered genes in NCM-460 cell line when treated with CN. Size represents the number of altered genes within the groups, indicated in red.

To confirm the safety profile of the treatments at a molecular level, we performed an overrepresentation analysis (ORA) to verify whether EU or CN affected pathways related to cell death or stress in normal colonocytes. According to the ORA, neither EU nor CN significantly altered genes linked to cell death or proliferation in the hallmark gene set, suggesting a non-toxic behavior of the two compounds on normal colonocytes (Figure 6 and Figure 7, Table 1 and Table 2).

### 2.6. SW-620 Overrepresentation Analysis

**Figure 8:**
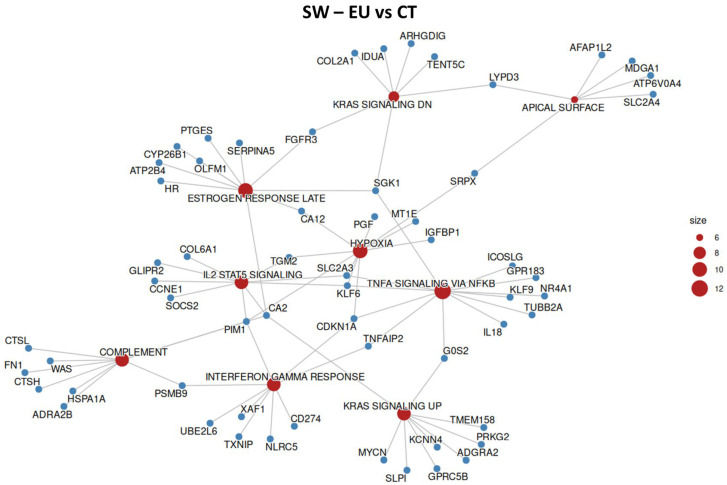
Overrepresented hallmark gene sets and respective altered genes in SW-620 cell line when treated with EU. Size represents the number of altered genes within the groups, indicated in red.

**Figure 9:**
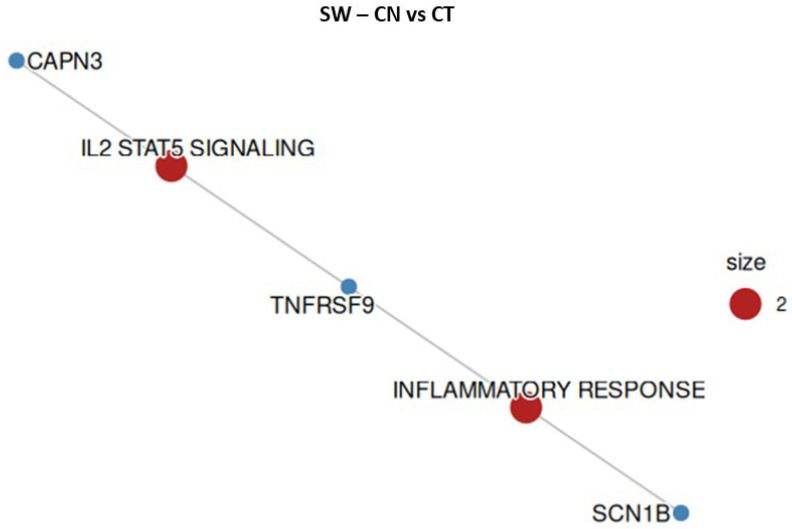
Overrepresented hallmark gene sets and respective altered genes in SW-620 cell line when treated with CN. Size represents the number of altered genes within the groups, indicated in red.

To understand the transcriptional changes observed in SW-620 cells treated with EU or CN, the ORA was employed to identify which hallmark genes were most affected by treatments. In SW-620 cells, EU treatment (Figure 8 and Table 3) affected genes linked to inflammatory signals (IL-2, STAT5, interferon-g response, TNF-a signaling via NF-kB), proliferation, survival and differentiation (KRAS signaling down, KRAS signaling up, hypoxia, estrogen response late, apical surface). Interestingly, CN treatment (Figure 9, Table 4) was confirmed to have a lower impact on the alteration of gene expression, inducing changes mainly linked to the inflammatory response pathways.

### 2.7. Caco-2 Overrepresentation Analysis

**Figure 10:**
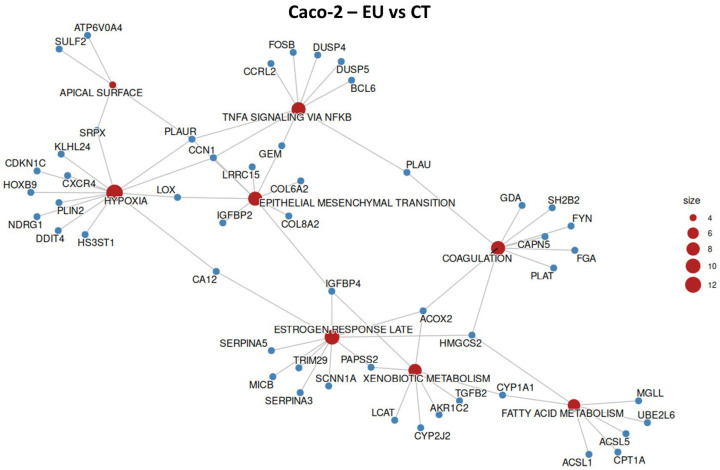
Overrepresented hallmark gene sets and respective altered genes in Caco-2 cell line treated with EU. Size represents the number of altered genes within the groups, indicated in red.

**Figure 11:**
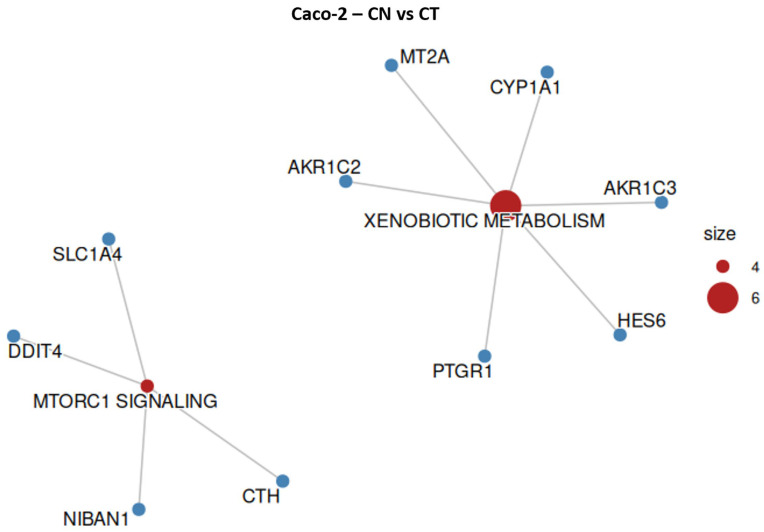
Overrepresented hallmark gene sets and respective altered genes in Caco-2 cell line when treated with CN. Size represents the number of altered genes within the groups, indicated in red.

The molecular pathways affected in Caco-2 cells treated with EU or CN were investigated through ORA. In Caco-2 cells, as well as SW-620, EU showed a stronger transcriptional effect with respect to CN. EU altered the expression of gene sets that are mainly related to tumor differentiation and angiogenesis (Figure 10, Table 5). Differently, CN significantly altered the expression of gene sets belonging to xenobiotic metabolism and MTORC1 signaling (Figure 11, Table 6). Xenobiotic metabolism genes were upregulated by both treatments, even if only two altered genes (*AKR1C2* and *CYP1A1*) were shared by CN and EU.

### 2.8. Western Blotting

**Figure 12:**
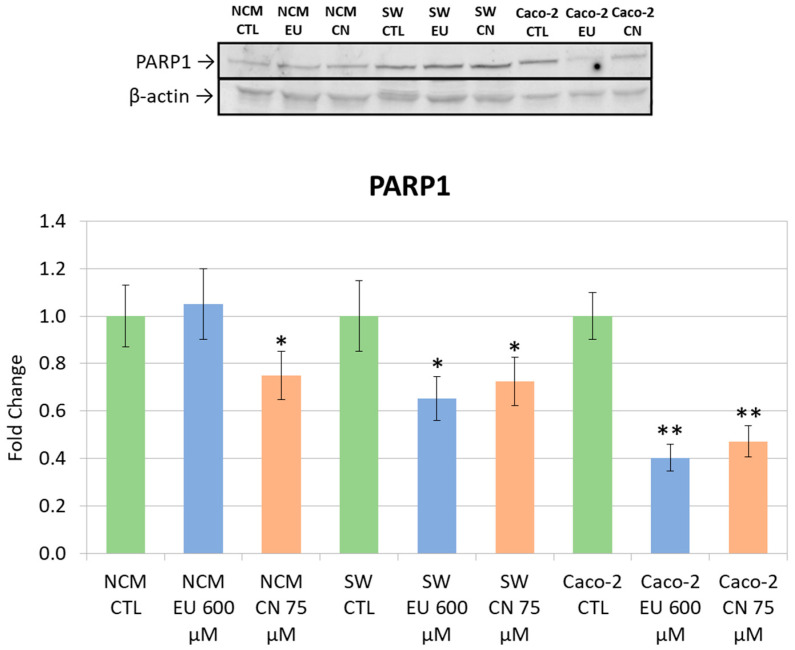
PARP1 and β-actin (reference control protein) expression (upper); densitometric quantification of WB bands (as PARP1/β-actin density) in the different lanes (lower). * *p* < 0.05 and ** *p* < 0.01 vs. CTL. Data represent the mean ± SEM of three independent experiments (n = 3).

To further investigate the molecular mechanisms responsible for growth inhibition, we analyzed the protein expression of key regulators of the cell cycle and apoptosis. Specifically, we evaluated p21 and PARP1 levels along with caspase-3 activation. PARP1 expression was analyzed in colonocytes following treatment with CN and EU. A significant downregulation of PARP1 levels was observed in NCM-460 cells following CN 75 μM treatment, while in both CRC cell lines (SW-620 and Caco-2) a consistent downregulation of PARP1 was detected with both treatments (Figure 12).

**Figure 13:**
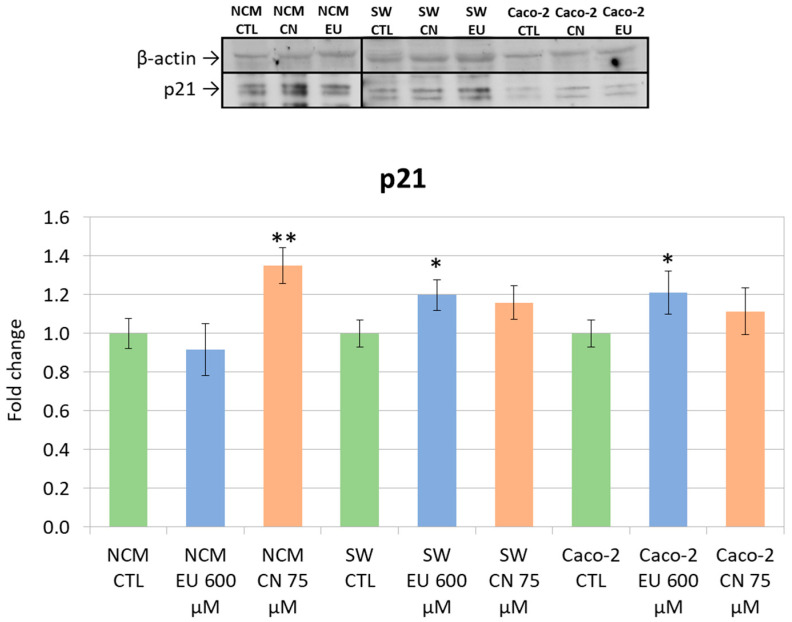
p21 and β-actin (reference control protein) expression (upper); densitometric quantification of WB bands (as p21/β-actin density) in the different lanes (lower). * *p<* 0.05 and ** *p* < 0.01 vs. CTL. Data represent the mean ± SEM of three independent experiments (n = 3).

In NCM-460 cells, a clear upregulation of p21 expression was observed but only upon treatment with CN 75μM. In CRC cell lines, p21 levels were significantly increased only by EU 600 μM treatment. (Figure 13)

**Figure 14:**
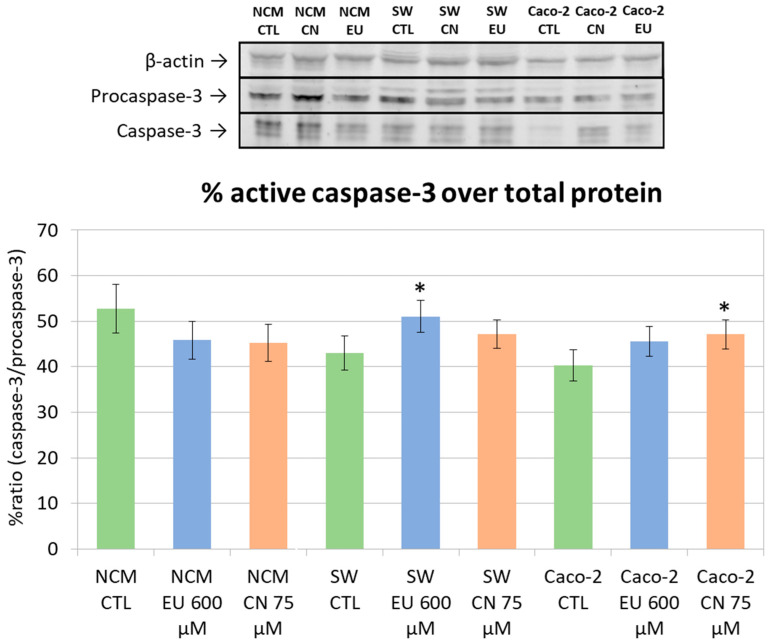
Procaspase-3, caspase-3, and β-actin (reference control protein) expression (upper); ratio between the densitometric quantification of WB bands of caspase-3 and procaspase-3 (as target/β-actin density) in the different lanes (lower). * *p* < 0.05 vs. CTL. Data represent the mean ± SEM of three independent experiments (n = 3).

The quantification of caspase-3 activation was calculated as the ratio between the active cleaved form of caspase-3 and the procaspase-3. In NCM-460 cells, treatment with EU or CN did not modify active caspase-3 compared to the control untreated cells. In SW-620 cells, only EU treatment (600 μM) induced an increase in active caspase-3 while in Caco-2 cells, only CN treatment (75 μM) increased caspase-3 activation with respect to the control (Figure 14).

## 3. Discussion

Several studies have demonstrated that EOs and their single components exhibit antitumor activities both in vitro and in vivo against various human cancers, either alone or in combination with anticancer drugs. CRC in particular has been identified as a target of these compounds [15]. In a previous study conducted by our research group, EU and CN demonstrated selective cytotoxic effects on Caco-2 and SW-620 cell lines, without affecting normal colon cells [16]. In this study, we wanted to gain a more comprehensive understanding of the effects of these compounds by dissecting the molecular mechanism by which EU and CN affect the viability of CRC cells. For this purpose, we chose the same tumor cell lines: Caco-2, quite differentiated and with a phenotype similar to that of enterocytes, characterized by strong barrier properties and a low basal inflammatory tone [17], and SW-620, derived from poorly differentiated metastatic adenocarcinomas, that exhibited higher activation of the NF-κB and STAT3 pathways [18]. The clonogenic assay was performed only in Caco-2 cells since they have the capability to form spheroids that mimic the architecture of the intestinal mucosa. These Caco-2 spheroids represent an excellent in vitro 3D model system capturing tissue-specific architecture and interactions with the extracellular matrix and therefore better recapitulate the complex in vivo environment [19]. The assay showed that while EU did not reduce the number of spheroids indicating inhibition of growth without significant cytotoxicity, CN exhibited a strong cytotoxic effect at both doses tested, completely inhibiting spheroid growth. On the other hand, when cell viability was analyzed in 2D monolayer cultures, EU showed the higher cytotoxic effects both in Caco-2 and SW-620 cell lines, consistent with its pro-apoptotic properties [20,21]. These differences could be explained by EU’s higher partition coefficient (logP = 2.49) [22], which may induce a “sink effect” where the compound sticks to the outer layer of the spheroids, resulting in a reduction in their size rather than in their number. In contrast, CN seems to have lower intrinsic cytotoxicity but is also less lipophilic and hydrophilic than EU (logP = 1.90) [23], and this characteristic may have allowed more homogeneous penetration within the spheroid structures. We tested the combination EU+ CN in 2D models to evaluate possible interactions since these two compounds can be both present in the same EO, such as that of cinnamon [24]. In this model, results showed a cytotoxicity of the mix comparable to that of single compounds, reflecting the predominance of EU’s activity, while the contribution of CN, mainly anti-inflammatory, appears limited under these conditions. These results suggest that, under the tested conditions, the combined effect reflected the independent activities of the two molecules in the absence of enhanced results compared to single treatments. Inflammation is associated with the onset and progression of CRC. In our study, we decided to test IL-4 and IL-8 expressions since these two cytokines have been involved in tumor onset and progression [25,26]. IL-4 was shown to promote tumor cell proliferation, survival, and metastasis by enhancing anti-apoptotic signals and contributing to a pro-tumor immune microenvironment, also contributing to therapy resistance [25]. IL-8 plays a pivotal role in CRC by promoting angiogenesis, metastasis, and tumor progression by stimulating epithelial–mesenchymal transition and by promoting cancer stem cell survival and chemoresistance [26]. EU and CN, individually and in combination, especially at higher concentrations, effectively reduced the levels of IL-4 and IL-8 in both CRC cell lines but with some differences. CN showed a strong efficacy in reducing IL-4 and IL-8 in both tumor cell lines, suggesting a stable action of the molecule on the pro-Th2 cytokine network. This effect is particularly evident since SW-620 cells are characterized by a strong activation of the NF-κB and STAT3 pathways [27], and CN has been shown to attenuate their activation by inhibiting IκBα phosphorylation and preventing the nuclear translocation of NF-κB p65, as well as by reducing JAK2-dependent STAT3 activation [28,29]. In contrast, EU does not primarily target inflammatory signaling but rather exerts cytotoxic effects probably through a mitochondria-dependent apoptotic mechanism, which becomes evident mainly at concentrations that induce ROS-mediated cell death [30]. Therefore, under conditions characterized by a high inflammatory tone (NF-κB- and STAT3-dependent), EU showed limited cytokine-modulatory capacity compared to CN. In Caco-2 cells, where the basal inflammatory tone is low, EU can activate the Nrf2–HO-1 pathway, a redox-regulated cytoprotective system that limits oxidative stress and restrains inflammatory signaling, leading to a moderate attenuation of cytokine release [31,32]. These mechanisms may account for the efficacy of EU on cytokine modulation in this cell line, characterized by a low basal inflammatory tone and a redox-regulated cytokine response, making them more sensitive to Nrf2–HO-1–mediated modulation rather than to inhibition of NF-κB/STAT3.

We investigated the molecular mechanisms of the action of EU and CN in Caco-2, SW-620, and NCM-460 cells through transcriptomic analysis. Subsequent in silico analysis of differentially expressed genes (DEGs) suggested that EU caused a stronger remodeling of gene expression compared to CN.

In Caco-2 cells, the coordinated downregulation of *SULF2*, *PLAUR*, *DUSP4*, and *ATP6V0A4* following EU exposure suggests a convergent suppression of pro-inflammatory and invasive programs. In fact, *SULF2* is involved in Wnt-driven tumor progression [33,34], *PLAUR* in extracellular matrix remodeling and migration [35,36], *DUSP4* in sustaining epithelial–mesenchymal plasticity [37,38], and *ATP6V0A4*/V-ATPase-dependent acidification in invasive phenotypes [39]. The transcriptional induction of *DDIT4* (*REDD1*) by CN points to the inhibition of growth-related pathways through the mTORC1–*DDIT4* axis. In particular, *DDIT4* promotes the suppression of mTORC1-dependent protein synthesis, thereby reducing anabolic metabolism and cellular growth [40,41]. Overall, although CN strongly suppressed IL-8 release in Caco-2 cells, its transcriptional impact was limited to xenobiotic metabolism and mTORC1 signaling. Conversely, EU induced a broader transcriptional remodeling affecting epithelial identity, extracellular matrix remodeling, and migratory behavior. The combined treatment therefore reflects complementary but not synergistic mechanisms, with EU predominantly driving apoptotic and phenotypic remodeling responses, and CN exerting a stronger influence on inflammatory signaling.

In SW-620 cells, EU treatment led to the downregulation of *MYCN*, *NR4A1*, and *SGK1*, a pattern that can be linked to a reduced activation of the proliferative, survival, and epithelial–mesenchymal transition (EMT) program. *MYCN* is known to support anabolic growth and cell survival [42,43], and its expression correlates with more aggressive behavior in CRC [44]. Aggressive subtypes of human CRC frequently exhibit amplification of the *c-myc* gene [45]; *NR4A1* has been linked to transcriptional programs that promote epithelial–mesenchymal plasticity and invasion [46,47], while *SGK1* is a downstream effector of PI3K/AKT that contributes to resistance to stress and to maintenance of EMT states [48,49]. Taken together, these data confirm that, in SW-620 cells, EU predominantly acts by attenuating survival and migration pathways and by reducing EMT-associated transcriptional signatures rather than by directly modulating inflammatory signaling.

CN instead was associated with the downregulation of *NR4A1* and *JUNB*, two transcriptional regulators involved in the expression of pro-inflammatory genes downstream of NF-κB and AP-1 [50]. This pattern is consistent with an attenuation of cytokine-related transcriptional activity, while the observed decrease in *TNFRSF9* is not related to a direct action on NF-κB/STAT pathways but may reflect a reduced stress- or immune-interaction signaling [51].

Western blotting was performed to fulfill the hypotheses generated from the transcriptomic analysis and to assess key protein expression in the different cell lines. As targets, we selected PARP1, p21 (*CDKN1A*), and caspase-3 as key downstream effectors of the regulatory networks identified rather than direct transcriptomic hits. Although these genes were not differentially expressed at the transcriptional level, we hypothesized that they could have been modulated at the protein level even in the absence of detectable transcriptional changes since protein activation and abundance often depend on post-transcriptional and post-translational regulations, which may account for discrepancies between mRNA and protein levels [52,53].

PARP1 is a key nuclear enzyme with a critical role in DNA damage response and serves as a major substrate for activated caspase-3. During apoptosis, caspase-3 cleaves the intact PARP1 (∼116 kDa) into its signature inactive fragment (∼89 kDa) [54]. The pronounced reduction in the full-length PARP1 band that we observed provides compelling evidence that the treatment successfully induced caspase activation and overcame the anti-apoptotic defensive response typical of cancer cells. This reduction is particularly relevant in CRC as the resistance to apoptosis is a primary driver of tumor progression and a common mechanism of therapy failure [55,56]. p21 (cyclin-dependent kinase inhibitor 1A or *CDKN1A*) acts as the principal inhibitor of cyclin-dependent kinases (CDKs) and regulates cell cycle progression through the G1 and G2 checkpoints [57]. Crucially, while p21 can initially mediate cell cycle arrest for DNA repair, its robust induction in tumor cells is considered a pivotal event that facilitates the transition toward senescence or apoptosis. In CRC cells, an increased p21 expression has been demonstrated to be essential for the induction of apoptosis and for overcoming resistance to various therapeutic agents [58,59]. Our Western blot analysis revealed a significant upregulation in p21 protein expression following treatment with EU and CN. This is a key finding as it is consistent with the enforcement of a cell cycle checkpoint that ultimately drives the cell towards programmed death. Therefore, the observed p21 upregulation supports our conclusion regarding the effective activation of the apoptotic pathways.

Caspase-3 is synthesized as the inactive zymogen, procaspase-3 (with a molecular mass of approximately ∼32 kDa), which requires proteolytic processing (cleavage) into its two active subunits (∼17 kDa and ∼19 kDa) in response to apoptotic stimuli [60]. The initiation of this processing cascade represents the definitive point of no return for apoptosis [61].

In the context of CRC, the ability to overcome apoptosis is a core mechanism of tumor progression and therapeutic failure. CRC cells frequently inhibit the activation of caspase-3 to ensure their survival. Consequently, treatment efficacy is in part related to its capacity to induce and activate caspase-3 [62]. Apoptosis activation is quantified by the ratio between processed (active) caspase-3 and procaspase-3 (inactive). An increase in this ratio serves as a critical indicator of committed apoptosis [63]. Our Western blot analysis revealed a marked increase in this ratio following treatment, indicating a substantial proteolytic activation of the caspase-3 into its mature, active form. As a quantitative indicator, the heightened caspase-3 ratio confirms a significant induction of apoptosis. The total reduction in PARP1 across all cell lines, despite the cell line-specific activation of caspase-3, suggests that EU and CN might also involve alternative pathways, such as caspase-7, or different apoptotic kinetics. These protein modulations were particularly evident at concentrations of 600 µM for EU and 75 µM for CN, correlating with the doses that inhibited cell viability. We observed an increase in caspase 3 activation that in SW620 followed EU treatment, while in Caco-2 followed CN treatment. These different behaviors are somehow expected since Caco-2 and SW620 represent different cellular models of CRC.

Regarding non-tumoral NCM-460 cells, PARP1 downregulation and the p21 increase in the absence of caspase-3 activation or cytotoxicity could be explained by the non-apoptotic roles of PARP1 (e.g., DNA repair and metabolic regulation) or could reflect adaptive or stress-related responses rather than apoptotic signaling.

This observation logically complements our other molecular findings (such as PARP1 cleavage and p21 upregulation), fostering the conclusion that EU treatment effectively triggered the enzymatic execution cascade of cell death, successfully circumventing the tumor’s intrinsic resistance mechanisms.

To better interpret these results, it is important to consider the study’s limitations. The experiments were conducted on stabilized cell lines, which, while recognized models of two distinct stages of CRC, cannot fully replicate the complex interactions between tumor cells, the extracellular matrix, the immune system, and the microbiota involved in CRC development in vivo. The effects of EU and CN should be studied in models that better reflect the genetic and tissue complexity of the disease. For example, studies on patients with rapid intestinal tumor development, such as those with familial adenomatous polyposis (FAP), could provide valuable insights [63]. Both EU and CN are natural compounds generally recognized as safe (GRAS) by the Food and Drug Administration (FDA) and European Food Safety Authority (EFSA); therefore, they are easily usable as natural molecules for CRC therapy. We are aware that in silico analysis as preliminary tools limited to providing a general overview of gene expression levels, which may not always correlate with protein synthesis. Additionally, while sequencing was performed on a pool of three biological replicates to ensure a representative signal, the lack of individual replicates may limit the statistical confidence in DEGs identification, despite the use of the NOISeq-Sim algorithm. Nevertheless, this study clearly defines the multitarget role of EU and CN, molecules capable of positively remodeling gene expression in CRC cells. Future studies focused on proteomics could confirm our findings or highlight new molecular targets underlying the anticancer activity of EU and CN.

## 4. Materials and Methods

### 4.1. Materials

Eugenol (EU) and cinnamaldehyde (CN) were provided by Xeda International S.A. (Saint-Andiol, France). EU pure (98% *w*/*w*, density = 1.06 g/mL, MW = 164.2 g/mol) and CN pure (98% *w*/*w*, density = 1.05 g/mL, MW = 132.16 g/mol) were stored at 4 °C and protected from light to maintain stability and prevent oxidation. CellTiter-Glo^®^ 2.0 Cell viability assay was purchased from Promega Corporation (Madison, WI, USA). Customized human magnetic Luminex^®^ screening assay was purchased from R&D Systems, Bio-Techne Corporation (Minneapolis, MN, USA). Ethanol absolute anhydrous was purchased from Carlo Erba (Milan, Italy). Lipopolysaccharide (LPS) was purchased from *E. coli* (strain 0111: B4, CliniSciences LTD, Nanterre, France). Quick Start™ Bradford 1× Dye Reagent, Immun-Blot^®^ PVDF Membrane, Prec Plus Protein Dual Color Stds 500#, XT MOPS Buffer Kit, 4–12% Criterion XT Bis-Tris Gel 18W 30 µ and transfer buffer were purchased from Bio-Rad (Hercules, CA, USA). RIPA buffer, Tris-buffered saline (TBS), Tween, phosphate buffered saline (PBS), Dulbecco’s modified Eagle’s medium-high glucose (DMEM-HG), fetal bovine serum (FBS), penicillin–streptomycin solution, L-Glutamine, protease inhibitors, and phosphatase inhibitors were purchased from Sigma Aldrich (Burlington, MA, USA).

### 4.2. Cell Cultures

The NCM-460 cell line was purchased from Bena Culture Collection (Beijing, China). Caco-2 and SW-620 cell lines were obtained from the American Type Culture Collection (ATCC) (Manassas, VA, USA). All the cells were cultured in high-glucose DMEM-HG containing 10% FBS, 2 mM L-glutamine, and antibiotics (100 U/mL penicillin and 100 µg/mL streptomycin) at 37 °C in a humidified incubator with 5% CO_2_, as previously described by Strillacci et al. [64].

### 4.3. Cell Treatments

Cells were seeded, in triplicate, in 96-well culture plates at 10^4^ cells/well for evaluation of the viability or in 24-well culture plates at 4 × 10^4^ cells/well for the cytokine analysis. After 24 h, medium was replaced with fresh ones containing different concentrations of EU and CN, alone or in combination: EU 600 and 300 µM; CN 75 and 37.5 µM; and their combinations (EU 600 µM + CN 75 µM and EU 300 + CN 37.5 µM). The selected concentrations are those that did not show cytotoxic activity on normal NCM-460 cells in our previous work, and their respective half [16]. Ethanol was used to solubilize essential oils. The final concentration of ethanol in the cell medium was kept lower than 0.1%. Ethanol 0.1% was added to the control (untreated) cells. To test the efficacy of our treatments in the modulation of LPS-induced inflammation, cells were cultured in complete medium containing LPS (1 µg/mL) alone or with the treatments under investigation for 24 h.

### 4.4. Clonogenic Cell Survival Assay

The antiproliferative effect on tumoral Caco-2 cells was evaluated using a clonogenic assay, following an adapted protocol based on the procedure described by Franken et al. [65]. Cells (0.8 × 10^3^ cells/well) were seeded into 6-well plates and incubated for 24 h. Subsequently, the cells were treated with varying concentrations of EU (EU; 600 µM and 300 µM) and CN (CN; 150 µM and 75 µM) dissolved in ethanol (final concentration < 0.1%) for 14 days, with media changes every 23 days. Untreated cells were used as controls. After the treatment period, cells were washed with PBS, fixed with paraformaldehyde (PFA), and stained with 0.1% crystal violet solution. Colony formation was analyzed under a microscope, and the number and size of spheroids per field was recorded. This assay was performed in three independent biological experiments (n = 3), with each condition analyzed in technical triplicate.

### 4.5. Cell Viability Assay

To evaluate the cytotoxic effects of our compounds, after treatments we performed the CellTiter-Glo^®^ 2.0 Cell Viability Assay according to the manufacturer’s protocol, and the plates were read in a microplate reader (Victor3™, Perkin Elmer, Waltham, MA, USA). Data are expressed as a percentage of viability when compared with untreated cells (negative control—considered as 100% viability). Positive controls were obtained by treating cells with 1% Triton X-100 (considered as 100% cell death). For background controls, blank wells with growth medium without cells were used. All viability tests were conducted as three independent biological experiments (n = 3), with each condition analyzed in technical triplicate.

### 4.6. Cytokine Analysis

To determine cytokines secreted by Caco-2, SW-620 and NCM-460 cells (IL-4 and IL-8) were measured using a multiplex Luminex^®^ reader (BioPlex 200) (Bio-Rad Laboratories Inc., Hercules, CA, USA) and a customized human magnetic Luminex^®^ screening assay, as previously described [66]. As a pro-inflammatory stimulus to increase cytokine secretion and mimic an intestinal pro-inflammatory condition, we added lipopolysaccharide (LPS) 5 μg/mL, a concentration widely used for these cell lines and devoid of toxicity.

For this assay, cells were seeded in 24-well culture plates at a density of 8 × 10^4^ cells/well. After the treatment period, the concentration of cytokines was based on an equivalent number of cells (at confluence they reach 250,000 cells per well) and on the evaluation of cell number and viability, as measured with the CellTiter-Glo^®^ 2.0 Cell Viability Assay. All experimental conditions were analyzed in three independent biological experiments (n = 3), each performed in technical quadruplicate.

### 4.7. Transcriptome Analysis

Once Caco-2, SW-620, and NCM-460 cells reached 70% confluence in a T75 flask, they were treated with 600 µM EU or 75 µM CN and incubated for 24 h. Untreated cells were used as controls. After incubation, both treated and untreated cells were harvested by trypsinization and centrifuged at 2500 rpm for 5 min, and the resulting cell pellet was collected. To ensure a representative transcriptional profile, cell pellets from three independent biological experiments were pooled together for each condition. Total RNA was then extracted from the pooled cell pellet and sent for RNA sequencing analysis. Consequently, sequencing was performed without further technical replicates. The sequencing analysis was conducted by Genomix4Life s.r.l. using the Illumina NovaSeq6000 platform (San Diego, CA, USA). The RNA-Seq analysis involved the following steps:

1. Quality control of the reads: Quality assessment of the raw sequencing data was performed using the FastQC tool, which calculated Phred scores to evaluate the probability of base-calling errors. Quality control metrics, such as per-base sequence quality, were summarized and visualized using the FastQC report [67].
2. Adapter and short read removal: Adapter sequences and reads shorter than 25 nucleotides were removed using the bioinformatics tool cutadapt (version 2.5) [68]. Although no adapter sequences were detected, this step was performed to eliminate very short reads and improve the overall quality of mapping.
3. Read alignment and quantification: Reads were aligned to the Homo sapiens reference genome (GRCh38.p13) [69] using STAR software (version 2.7.10b) [70]. The alignment was performed using default settings for paired-end reads, and multi-mapping and gap tolerance parameters were kept at their default values. Transcript quantification was performed using the FeatureCounts algorithm to calculate read counts mapped to genomic features, including genes and exons.
4. Differential expression analysis: R software (version 4.5.0) was used to create a matrix of all expressed transcripts with corresponding read counts. Data were normalized using the RSEM method to ensure consistency across samples. Differentially expressed genes were identified using the Bioconductor package NOISeq-Sim, a non-parametric method suitable for nonreplicated samples [71].

#### 4.7.1. Criteria for Differential Gene Selection

Differentially expressed genes with a probability (P) > 0.85 of being significantly different and with a fold change > 1.5 were selected for further analysis. This probability threshold (P) represents the posterior probability of differential expression—a characteristic metric of the NOISeq algorithm—and does not correspond to a traditional *p*-value [71]. Genes were compared between treated (600 µM EU or 75 µM CN) and untreated cells.

#### 4.7.2. Overrepresentation Analysis (ORA)

To assess the differentially expressed cellular metabolic pathway, an overrepresentation analysis was performed using the R/ClusterProfiler v4.16.0 package [72] and the Hallmark gene set (collection: H) from the Human MSigDb Collections [73]. This collection represents well-defined biological states and cellular processes. A *p*-value cutoff of 0.05 was applied to determine statistical relevance, and the Benjamini–Hochberg method was adopted for multiple-testing correction, allowing for further filtering for q-values < 0.10.

### 4.8. Western Blotting

Once Caco-2, SW-620, and NCM-460 cells reached 70% confluence in a T75 flask, they were treated with 600 µM EU or 75 µM CN and incubated for 24 h. Untreated cells were used as controls. After treatment, cells were washed with phosphate-buffered saline (PBS), detached using a cell scraper, and collected in 15 mL Falcon tubes. The samples were then centrifuged at 1100 rpm for 5 min at 4 °C, and the cell pellets were resuspended in 100 µL of RIPA lysis buffer containing protease and phosphatase inhibitors. After vortexing for 3–5 min, lysates were incubated on ice for 30 min and then centrifuged at 16,000× *g* for 30 min at 4 °C. The supernatants were aliquoted and stored at −80 °C. Protein concentrations were determined using the Bradford assay following manufacturer instructions. A total of 50 µg of protein per sample was separated by SDS-PAGE on a 4–12% polyacrylamide precasted gel (Bio-Rad). The separated proteins were transferred onto PVDF membranes using the Trans-Blot Turbo Transfer System (Bio-Rad), following the manufacturer’s protocol. Membranes were blocked with 5% bovine serum albumin (BSA) in Tris-buffered saline with 0.1% Tween-20 (TBS-T) for 1 h at room temperature and incubated overnight at 4 °C with the following primary antibodies: anti-β-actin (1:1000, Sigma-Aldrich), anti-p21, anti-PARP1, anti-procaspase3, and anti-caspase-3 (1:1000, Invitrogen, Waltham, MA, USA). After washing, membranes were incubated with the appropriate secondary antibodies (cyanine5 or cyanine3 anti-rabbit and anti-mouse, Invitrogen) at a dilution of 1:2500 for 1 h in the dark. Membranes were washed three times with TBS-T and visualized using the Bio-Rad ChemiDoc™ MP imaging system (Bio-Rad, Hercules, CA, USA). Densitometric analysis of band intensity was performed using Image Lab software (version 5.2.1), and values were normalized to β-actin as the loading control. This analysis was performed on three independent biological replicates (n = 3), with each sample loaded in single for each experiment.

### 4.9. Statistical Analysis

All data were analyzed using the software GraphPad Prism v.9.00 (GraphPad Soft-ware, San Diego, CA, USA). Quantitative variables were expressed as means ± SEM. Statistical analysis was performed using a Student’s *t*-test and one-way and two-way ANOVAs, using the unpaired comparison and the multiple comparisons tests Tukey and Dunnett, respectively. Values of *p* < 0.05, *p* < 0.01, *p* < 0.001 and *p* < 0.0001 were considered significant, with different strengths of evidence.

## 5. Conclusions

The literature on the antitumor effects of eugenol (EU) and cinnamaldehyde (CN) is extensive; nevertheless, little was known regarding the molecular targets of these two molecules. This study addresses this specific scientific question for the first time, highlighting the multitarget mechanisms of action of EU and CN, and their main target genes. This lays the groundwork for future studies that could utilize these two molecules in CRC therapy.

## Figures and Tables

**Table 1:** Overrepresented hallmark gene sets, fold enrichment, and respective altered genes in NCM-460 cell line when treated with EU.

Gene Count	Genes	Fold Enrichment	Hallmark Gene Set
7	*F10/PTGES/ABCC2/MT2A/* *FABP1/PDK4/CYP1A1*	3.066	Xenobiotic Metabolism
7	*CTSE/F10/C8G/MEP1A/* *RAPGEF3/CRIP2/HMGCS2*	5.44	Coagulation
6	*ACSL1/AQP7/FABP1/* *HMGCS1/HMGCS2/CYP1A1*	3.069	Fatty Acid Metabolism

**Table 2:** Overrepresented hallmark gene sets, fold enrichment, and respective altered genes in NCM-460 cell line when treated with CN.

Gene Count	Genes	Fold Enrichment	Hallmark Gene Set
2	*CYP1A1/FOSB*	5.746	UV Response Up
3	*CYP1A1/ALDH1A1/* *ALDH3A1*	8.619	Fatty Acid Metabolism

**Table 3:** Overrepresented hallmark gene sets, fold enrichment, and respective altered genes in SW-620 cell line when treated with EU.

Gene Count	Genes	Fold Enrichment	Hallmark Gene Set
6	*AFAP1L2/LYPD3/ATP6V0A4/SRPX/* *SLC2A4/MDGA1*	6.966	Apical surface
12	*ICOSLG/TUBB2A/G0S2/CDKN1A/* *TNFAIP2/GPR183/KLF9/IL18/KLF6/* *NR4A1/SLC2A3/SGK1*	2.896	TNF-α Signaling via NF-kB
10	*FGFR3/ATP2B4/SERPINA5/CA2/* *OLFM1/CA12/HR/CYP26B1/PTGES/* *SGK1*	2.413	Estrogen Response Late
10	*TGM2/CDKN1A/CA12/IGFBP1/PGF/* *MT1E/SRPX/PIM1/KLF6/SLC2A3*	2.334	Hypoxia
9	*SLPI/G0S2/ADGRA2/PRKG2/CA2/* *MYCN/TMEM158/KCNN4/GPRC5B*	2.447	KRAS Signaling Up
9	*CA2/PSMB9/HSPA1A/ADRA2B/FN1/* *PIM1/CTSH/CTSL/WAS*	2.431	Complement
9	*TXNIP/PSMB9/CDKN1A/TNFAIP2/* *PIM1/XAF1/NLRC5/UBE2L6/CD274*	2.431	Interferon-γ response
9	*COL6A1/SOCS2/TGM2/CA2/GLIPR2/* *CCNE1/PIM1/KLF6/SLC2A3*	2.287	IL2 STAT5 Signaling
7	*FGFR3/ARHGDIG/LYPD3/COL2A1/* *IDUA/TENT5C/SGK1*	2.425	KRAS Signaling Down

**Table 4:** Overrepresented hallmark gene sets, fold enrichment, and respective altered genes in SW-620 cell line when treated with CN.

Gene Count	Genes	Fold Enrichment	Hallmark Gene Set
2	*TNFRSF9/SCN1B*	6.844	Inflammatory Response
2	*TNFRSF9/CAPN3*	5.507	IL-2 STAT5 Signaling

**Table 5:** Overrepresented hallmark gene sets, fold enrichment, and respective altered genes in Caco-2 cell line when treated with EU.

Gene Count	Genes	Fold Enrichment	Hallmark Gene Set
13	*CXCR4/CDKN1C/HS3ST1/* *CCN1/PLIN2/NDRG1/PLAUR*	3.274	Hypoxia
9	*SH2B2/CAPN5/ACOX2/FGA/* *GDA/PLAU/FYN/HMGCS2/* *PLAT*	0.384	Coagulation
10	*SERPINA3/MICB/PAPSS2/* *SERPINA5/SCNN1A/ACOX2/* *IGFBP4/CA12/TRIM29/* *HMGCS2*	2.59	Estrogen Response Late
9	*LRRC15/COL8A2/IGFBP2/* *CCN1/GEM/IGFBP4/COL6A2/* *PLAUR/LOX*	2.597	Mesenchymal Transition
4	*ATP6V0A4/SULF2/PLAUR/* *SRPX*	5.065	Apical Surface
9	*CCRL2/DUSP4/BCL6/CCN1/* *GEM/* *DUSP5/PLAU/PLAUR/FOSB*	2.292	TNF-α Signaling via NF-kB
8	*TGFB2/CYP2J2/PAPSS2/LCAT/* *ACOX2/AKR1C2/IGFBP4/* *CYP1A1*	2.158	Xenobiotic Metabolism
7	*UBE2L6/ACSL1/ACSL5/CPT1A/MGLL/HMGCS2/CYP1A1*	2.263	Fatty Acid Metabolism

**Table 6:** Overrepresented hallmark gene sets, fold enrichment, and respective altered genes in Caco-2 cell line when treated with CN.

Gene Count	Genes	Fold Enrichment	Hallmark Gene Set
6	*HES6/PTGR1/AKR1C3/* *MT2A/CYP1A1/AKR1C2*	6.272	Xenobiotic Metabolism
4	*DDIT4/NIBAN1/* *CTH/SLC1A4*	3.664	MTORC1 Signaling

## Data Availability

The data sets generated during the current study are available from the corresponding author upon reasonable request.
